# Defect of LSS Disrupts Lens Development in Cataractogenesis

**DOI:** 10.3389/fcell.2021.788422

**Published:** 2021-12-02

**Authors:** Minglei Zhao, Tingfang Mei, Bizhi Shang, Bin Zou, Qing Lian, Wenchang Xu, Keling Wu, Yuhua Lai, Chujun Liu, Lai Wei, Jie Zhu, Kang Zhang, Yizhi Liu, Ling Zhao

**Affiliations:** ^1^ State Key Laboratory of Ophthalmology, Zhongshan Ophthalmic Center, Sun Yat-sen University, Guangdong Provincial Key Laboratory of Ophthalmology and Visual Science, Guangzhou, China; ^2^ Guangdong Province Key Laboratory of Brain Function and Disease, Zhongshan School of Medicine, Sun Yat-sen University, Guangzhou, China; ^3^ Dongguan Guangming Ophthalmic Hospital, Dongguan, China; ^4^ Guangzhou Women and Children’s Medical Center, Guangzhou Medical University, Guangzhou, China; ^5^ Center for Biomedicine and Innovations, Faculty of Medicine, Macau University of Science and Technology and University Hospital, Macau, China; ^6^ Guangzhou Regenerative Medicine and Health Guangdong Laboratory, Guangzhou, China; ^7^ Research Unit of Ocular Development and Regeneration, Chinese Academy of Medical Sciences, Guangzhou, China

**Keywords:** lanosterol synthase, congenital cataract, mouse model, mutation, lens development

## Abstract

Congenital cataract is one of the leading causes of blindness in children worldwide. About one-third of congenital cataracts are caused by genetic defects. LSS, which encodes lanosterol synthase, is a causal gene for congenital cataracts. LSS is critical in preventing abnormal protein aggregation of various cataract-causing mutant crystallins; however, its roles in lens development remain largely unknown. In our study, we generated a mouse model harboring Lss G589S mutation, which is homologous to cataract-causing G588S mutation in human LSS. Lss^G589S/G589S^ mice exhibited neonatal lethality at postal day 0 (P0), whereas these mice showed severe opacity in eye lens. Also, we found that cataract was formed at E17.5 after we examined the opacity of embryonic lens from E13.5 to E18.5. Moreover, disrupted lens differentiation occurred at E14.5 prior to formation of the opacity of eye lens, shown as delayed differentiation of lens secondary fiber and disordered lens fiber organization. In addition, RNA-seq analysis indicated that cholesterol synthesis signaling pathways were significantly downregulated. Overall, our findings provide clear evidence that a mouse model harboring a homozygous Lss G589S mutation can recapitulate human congenital cataract. Our study points out that LSS functions as a critical determinant of lens development, which will contribute to better understanding LSS defects in cataractogenesis and developing therapies for cataracts.

## Introduction

Cataracts occur due to a loss of transparency in the crystalline lens of the eye, which is the most leading cause of blindness and impaired vision worldwide (Liu et al., 2017). Up to one-third of congenital cataracts (CC) are inherited (Hejtmancik, 2008). There are more than 100 genes that have been reported to cause CC (Messina-Baas and Cuevas-Covarrubias, 2017). In known cataract causal genes, half of them belong to the crystallin family. Mutations in genes encoding membrane transport, scaffolding proteins, transcription factors, heat shock transcription factor, and metabolism-related proteins are also identified to cause CC (Shiels and Hejtmancik, 2013; Zhao et al., 2015; Sun et al., 2019).

LSS, which encodes lanosterol synthase, is a causal gene for CC (Zhao et al., 2015; Chen and Liu, 2017). LSS is a key early rate-limiting enzyme in the biosynthesis of four-ringed steroid structure intermediate products including cholesterol, steroid hormones, and vitamin D (Huff and Telford, 2005). LSS is critical in preventing abnormal crystallin protein aggregation (Zhao et al., 2015). In our previous study, two distinct homozygous LSS missense mutations (W581R and G588S) impaired key catalytic functions of LSS and were identified in two families with extensive CC (Zhao et al., 2015). Although LSS mutations with an additional mutation in Fdft1 caused cholesterol deficiency-associated cataracts in the Shumiya cataract rat (SCR) and lens-specific Lss knockout mice had cataracts, SCRs developed mature cataracts at around 11 weeks of age and cataracts were shown in lens-specific Lss knockout mice at 14 weeks of age (Mori et al., 2006; Wada et al., 2020). Thus, the roles of LSS in early-stage of lens development remain largely unknown.

In our study, we generated a knock-in mouse model with G589S mutation in Lss, which is homologous to cataract-causing G588S mutation in human LSS. Our finding showed that mice harboring biallelic Lss G589S mutations exhibited CC at E17.5 and disrupted lens fiber differentiation at E14.5. Taken together, our study points out that LSS is a critical determinant in lens development, which will contribute to better understanding of the roles of LSS in cataractogenesis.

## Materials and methods

### Animals

**FIGURE 1:**
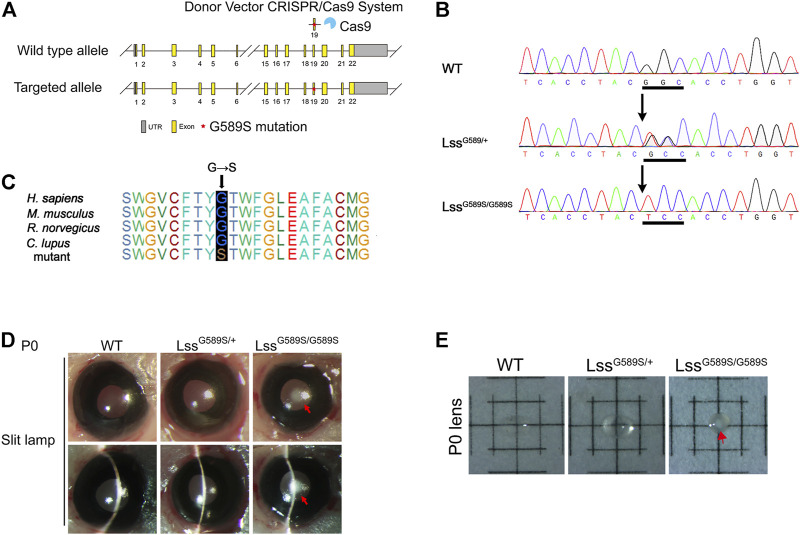
The lens of engineered Lss^G589S/G589S^ mice could recapitulate the phenotype of human congenital cataract. **(A)** Schematic diagram of Lss G589S knock-in mice generated at exon 19 of Lss *via* the CRISPR/Cas9 system. **(B)** Sanger sequencing performed to validate the G589S missense mutation (GGC > TCC) in WT, Lss^G589S/+^, and Lss^G589S/G589S^ mice. Underlined sequences indicate the changed nucleic acids. **(C)** The amino acid residue G588 in human LSS is highly conserved across species, which is homologous to mouse G589. Blue box refers to the homologous site of LSS G588 in *Homo sapiens*, *Mus musculus*, *Rattus norvegicus*, and *Canis lupus*. **(D)** Mouse lens were examined by slit lamp. Red arrows indicated congenital cataract formed in lens of Lss^G589S/G589S^ mice at P0. **(E)** Lenses were isolated and photographed by a dissecting microscope. Red arrows indicated cataract formed in the lens of Lss^G589S/G589S^ mice at P0.

All the animal procedures were approved by the Animal Ethical Committee at Zhongshan Ophthalmic Center, Sun Yat-sen University (Guangzhou, China), and all the uses of animals were performed in accordance with the Association for Research in Vision and Ophthalmology (ARVO) statement. C57BL/6J wild-type (WT) mice and Lss G589S knock-in (KI) mice produced on the C57BL/6J background were obtained from GemPharmatech Co., Ltd. (Jiangsu, China). The G589S mutation of mouse Lss, homologous to cataract-causing G588S mutation in human LSS, was generated by CRISPR/Cas9-mediated genome editing in GemPharmatech Co., Ltd. (Nanjing, China) (Figure 1A). Mice with Lss G589S mutation were genotyped by Sanger sequencing using PCR of tail genomic DNA. PCR primers are 5′-GCC​TTA​GCC​CAG​TGC​TAG​GAA​T-3′ and 5′-CAT​GGT​TTC​TGC​TTC​AGT​TCC​T-3′. To collect mouse embryos at various stages, the day a vaginal plug was observed after mating was designated as embryonic day 0.5 (E0.5). Mouse lens at postnatal day 0 (P0) and E13.5-E18.5 were collected for further studies.

### Slit-lamp photography and lens photography

The eyelids of neonatal mice at P0 were removed immediately. A photo was taken on the eyes under a slit lamp with appropriate angle and light intensity under the slit lamp. Lens were enucleated, immersed in lens medium (Medium 199, Earle’s Salts, Thermo), and photos were taken immediately under dissection microscopy (M205 FA, Leica, Wetzlar, Germany).

### Histology

Histology was performed as previously described (Lian et al., 2019). Enucleated eyeballs from P0 mice and embryos from Lss^G589S/G589S^, Lss^G589S/+^, and WT mice were fixed with 4% paraformaldehyde overnight at 4°C, dehydrated in a series of ethanol with ascending concentrations, cleared in xylene, and embedded in paraffin. The tissues were sectioned in a vertical pupillary optic nerve plane and stained with hematoxylin and eosin. The images were captured using a TissueFAXS microscope (TissueGnostics, Vienna, Austria).

### Immunofluorescence

For frozen sections, enucleated eyeballs from P0 mice and embryos were fixed in 4% paraformaldehyde for 30 min at room temperature and 30% sucrose dehydrated over night at 4°C, then embedded in OCT, frozen, and sectioned at 10 µm. Cryosections were permeabilized in 0.5% Triton X-100/PBS for 2 min, blocked in 5% donkey serum and 5% BSA in PBS for 1 h at room temperature, then incubated with primary antibodies overnight at 4°C. After rinsing, the sections were incubated with fluorescence-labeled secondary antibodies (Alexa Fluor 488, or Alexa Fluor 546, Invitrogen, Carlsbad, CA, USA) for 2 h at room temperature and counterstained with DAPI (1:1,000, H-1200, Vector Labs, Burlingame, CA, USA). Primary antibodies used for immunofluorescence were LSS (1:300, 18693-1-AP, Proteintech, Wuhan, China), p57^KIP2^ (1:200, ab75974, Abcam, Cambridge, MA, USA), Pax6 (1:200, PRB-278P, BioLegend, San Diego, CA, USA), and Prox1 (1:200, 11067-2-AP, Proteintech). The images were captured with an LSM980 confocal scanning microscope (Carl Zeiss, Thornwood, NY, USA) or a TissueFAXS microscope (TissueGnostics, Austria).

### Transmission electron microscopy

Mouse lens at P0 were fixed and processed as previously described (Morishita et al., 2013). Lenses were immediately placed into fixative consisting of 2.5% glutaraldehyde and 2% formaldehyde in 0.1 mol/l cacodylate buffer with 0.08 mol/l CaCl_2_ at 4°C. Then, the lens was postfixed for 1.5 h in 1% aqueous OsO_4_, dehydrated through graded acetone, transitioned in propylene oxide, infiltrated with propylene oxide and EPON mixtures (TAAB 812 resin; Marivac, Quebec, QC, Canada), embedded in EPON, and cured for 48 h at 60°C. One-micron lens sections across the equatorial plane were collected on glass slides and stained with toluidine-blue in 1% borate buffer. Thin sections were cut at 80-100 nm and stained with saturated, aqueous uranyl acetate, and Sato’s lead stain and imaged with a transmission electron microscope (Tecnai G2 Spirit, FEI, Hillsboro, OR, Czech).

### RNA-seq analysis

RNA sequencing and analysis were performed by Berry Genomics Corporation, Beijing, China. Lenses were isolated from Lss^G589S/G589S^, Lss^G589S/+^, and WT mice embryos at E14.5 and three biological replicates from Lss^G589S/G589S^, Lss^G589S/+^, and WT, each replicate consisting of nine pups (18 lenses) at E14.5 (Anand et al., 2018). Lenses were dissolved immediately in TRIzol reagent (Invitrogen, USA), and total RNA was extracted according to the manufacturer’s instructions (Khan et al., 2015). A total amount of 1 µg RNA per sample was used as input material. Sequencing libraries were generated using NEBNext^®^ Ultra™ RNA Library Prep Kit for Illumina^®^ (NEB, USA) following the manufacturer’s recommendations, and index codes were added to attribute sequences to each sample. The clustering of the index-coded samples was performed on a cBot Cluster Generation System using TruSeq PE Cluster Kit v3-cBot-HS (Illumina, San Diego, CA, USA) according to the manufacturer’s instructions. After cluster generation, the library preparations were sequenced on an Illumina NovaSeq platform and 150-bp paired-end reads were generated. Clean data with high quality after processed raw data were aligned to the mouse reference genome (GRCm38/mm10) using TopHat v2.0.12. After applying the HTSeq v0.6.1 to extract the raw count tables based on the aligned bam files, DESeq2111 was used to perform normalization and differential gene expression analysis. Ingenuity pathway analysis (IPA) (Qiagen Inc., Hilden, Germany) software was applied to analyze signaling pathways and differentially expressed genes (adjusted *p*-value < 0.05).

### Western blot

Western blot was performed as previously described (Wu et al., 2020). Two isolated lenses from one embryo at E14.5 were homogenized and lysed in lysis buffer (RIPA, protease inhibitors, and PMSF mixture, pH 7.6) on ice for 10 min, and the lysates were centrifuged at 13,000 rpm for 20 min. Each group had three biological replicates. Lens lysates were separated by 10% SDS-PAGE and transferred to PVDF (0.2 mm). Membranes were blotted with 5% milk (fat free) prepared in Tris-buffered saline Plus 0.1% Tween-20 (TBST) at room temperature for 1 h and incubated with primary antibodies diluted in 5% milk overnight at 4°C. Blots were visualized using secondary antibodies by enhanced chemiluminescence (Thermo, Waltham, MA, USA). Primary antibodies used for Western blotting were LSS (18693-1-AP, Proteintech) and tubulin (2146, CST). ImageJ (NIH, Bethesda, MD, USA) was applied in Western blotting analysis.

### Statistics

The data are presented as mean ± SEM. Student’s t test was applied to determine statistical significance. Statistical significance was defined as *p* < 0.05.

## Results

### The mice harboring a homozygous mutation (Lss^G589S/G589S^) can recapitulate human CC

Based on the evidence of LSS gene mutational analysis in human CC (Zhao et al., 2015; Chen and Liu, 2017), we generated a mouse model harboring Lss G589S mutation, which is homologous to cataract-causing G588S mutation in human LSS. Targeted alleles were introduced in exon 19 of Lss on mouse chromosome 10 *via* CRISPR/Cas9 system (Figure 1A). Sanger sequencing was performed to validate the G589S missense mutation (GGC > TCC) in Lss (Figure 1B). The amino acid residue G588 in human LSS is highly conserved across species and homologous to mouse G589 (Figure 1C). Mouse lenses were examined by slit-lamp and photographed by a dissecting microscope. WT and Lss^G589S/+^ mice showed transparent lenses under a slit lamp (Figure 1D) and dissecting microscope (Figure 1E). Compared to WT and Lss^G589S/+^ mice, Lss^G589S/G589S^ showed severe opacity of eye lens at postal day 0 (P0), whereas these mice exhibited neonatal lethality at P0 (Figure 1D). The cataract plaque was mainly located in the nucleus of lens (Figure 1E). Another line of Lss^G589S/G589S^ mice also showed the same phenotypes with CC (Supplemental Figure 1A,B) and died at P0. Thus, our finding showed that a mouse model harboring an Lss G589S mutation was generated and the mice harboring a biallelic mutation of Lss G589S exhibited CC.

### Disrupted lens structure caused by a homozygous Lss^G589S/G589S^ mutation at P0

**FIGURE 2:**
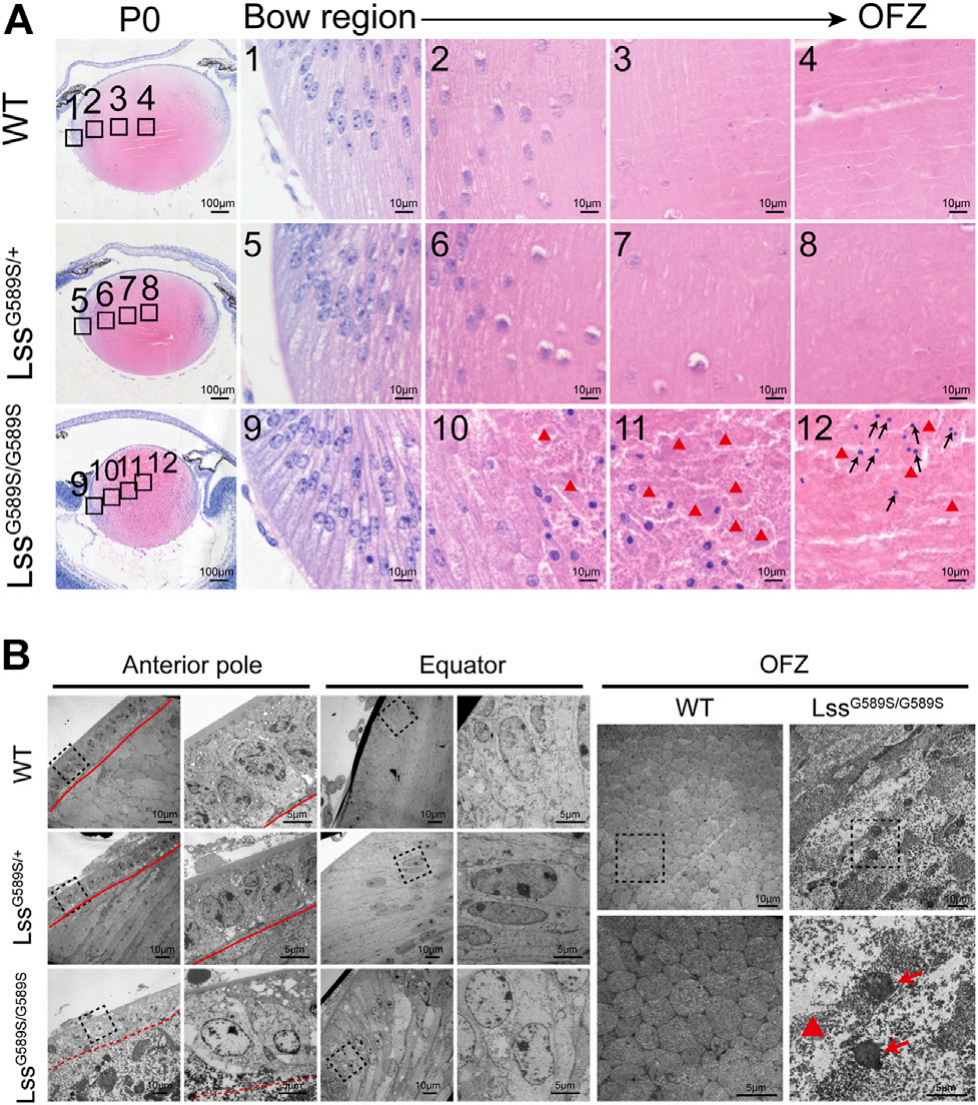
Disrupted lens structure caused by a homozygous Lss G589S mutation at P0. **(A)** Representative hematoxylin and eosin staining images of lens structure in WT, Lss^G589S/+^, and Lss^G589S/G589S^ mice at P0. Boxes of numbers 1–4, 5-8, and 9–12 indicate four continuous and comparable zones from the lens equator to OFZ within the lens fiber cell regions of WT, Lss^G589S/+^, and Lss^G589S/G589S^, respectively (the leftmost panel, black boxes). Scale bar: 100 μm. The enlargements are the magnifications of the boxed inset shown in the left panel (numbers 1–4, 5–8, and 9–12). Scale bar: 10 μm. Red triangles indicate debris and bulks of fibers in Lss^G589S/G589S^ mouse lens. Black arrows indicate that a large number of fiber cell nuclei were still retained at the OFZ region of Lss^G589S/G589S^ mouse lens. **(B)** Electron micrographs of lens morphology at the anterior pole, equator, and OFZ regions in WT, Lss^G589S/+^, and Lss^G589S/G589S^ mice. *n* = 4 lenses per group. At the anterior pole region, representative images of the lens epithelial–fiber interface (EFI) were shown. Red solid lines indicate lens epithelial–fiber interface (EFI) in WT and Lss^G589S/+^ mice. Red dashed line indicates lens EFI in Lss^G589S/G589S^ mice. Boxes indicate morphology of lens epithelial cells and EFI in WT, Lss^G589S/+^, and Lss^G589S/G589S^ mice **(left panel)**. Scale bar: 10 μm. The enlargement is the magnification of the boxed inset shown in the left panel. Scale bar: 5 μm. At the equator region, representative images of alignment and morphology of secondary fiber cells were shown. Boxes indicate the morphology of secondary fiber cells **(left panel)**. Scale bar: 10 μm. The enlargement is the magnification of the boxed inset shown in the left panel. Scale bar: 5 μm. At the OFZ region, representative images of alignment and morphology of fiber cells were shown. Scale bar: 10 μm. The enlargement is the magnification of the boxed inset shown in the upper panel. Red triangle indicates high-density deposits of fiber debris, and red arrows indicate condensed nuclei in the central OFZ of Lss^G589S/G589S^ lens. Scale bar: 5 μm.

The pups with a homozygous Lss^G589S/G589S^ mutation were born with abnormally small lenses with severely structural defects at P0, while the lens structure is normal in WT and Lss^G589S/+^ mice at P0 (Figure 2A). The process of denucleation and organization of lens fibers was investigated from the lens bow region to the organelle-free zone (OFZ) in WT, Lss^G589S/+^, and Lss^G589S/G589S^ mice (Figure 2A). Our results showed that the process of denucleation had been completed at OFZ and the lens fiber was normally arranged in WT and Lss^G589S/+^ mouse lens (Figure 2A 1-4, Figure 2A 5-8). However, in the lens of Lss^G589S/G589S^ mice, the process of denucleation failed to complete at OFZ (Figure 2A 9-12) and a large number of fiber cell nuclei were still retained at the OFZ region (Figure 2A 12, black arrows). Also, compared with WT and Lss^G589S/+^ mouse lens, the fiber nuclei at the OFZ region were more highly condensed in the lens of Lss^G589S/G589S^ mice. Moreover, debris and bulks of fibers were clearly observed in the lens of Lss^G589S/G589S^ mice (Figure 2A 10-12, red triangles).

High-resolution structural features of lens were provided by transmission electron microscopy (TEM) examination. A monolayer of anterior lens epithelial cells and well-aligned lens fiber cells at the anterior pole were shown in the lens of WT and Lss^G589S/+^ mice (Figure 2B, Supplementary Figure 2A). Also, the epithelial–fiber interface (EFI) was intact in WT and Lss^G589S/+^ lens (Figure 2B, Supplementary Figure 2A, red solid lines), while the EFI was severely disrupted in Lss^G589S/G589S^ homozygous lens (Figure 2B, Supplementary Figure 2A, red dashed line). At the lens equator, secondary fiber cells were well organized with elongated shape in WT and Lss^G589S/+^ heterozygous lens, while secondary fiber cells were disorganized in alignment with the swollen shape in Lss^G589S/G589S^ lens (Figure 2B). In addition, compared with WT mice, high-density deposits of fiber debris (Figure 2B, Supplementary Figure 2B, red triangle) and condensed nuclei (Figure 2B, red arrows, Supplementary Figure 2B) were observed in the central organelle-free zone (OFZ) of Lss^G589S/G589S^ lens.

### Different distribution of LSS protein in lens epithelial cells at P0

**FIGURE 3:**
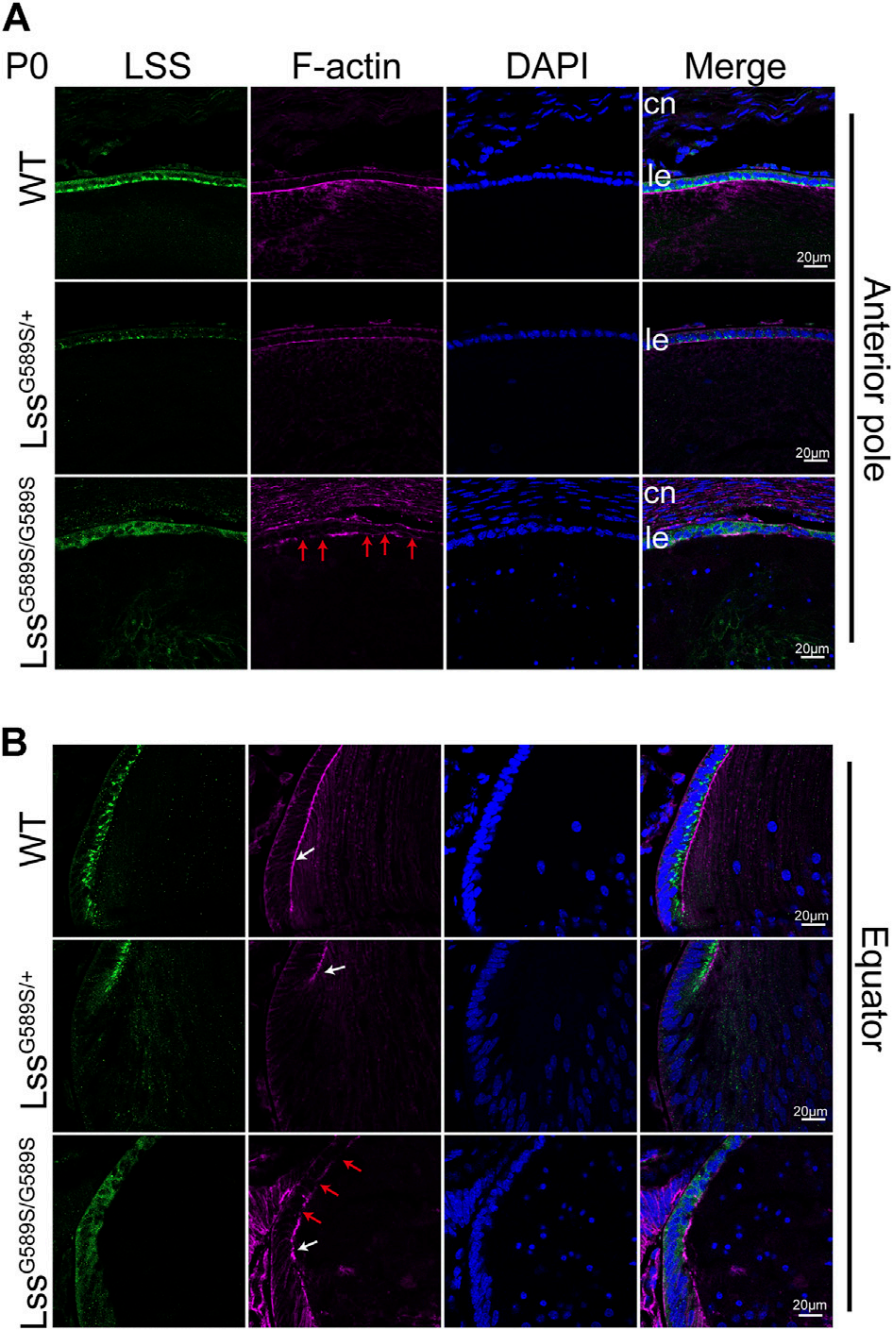
Different distributions of LSS protein in lens epithelial cells at P0. **(A)** The expression of LSS and its distribution in lens epithelial cells at the anterior pole region of WT, Lss^G589S/+^, and Lss^G589S/G589S^ mice at P0. **(B)** The expression of LSS and its distribution in lens epithelial cells at the equator region. White arrows indicate EFI, and red arrows indicate discontinuous EFI stained by F-actin. cn, cornea. Le, lens epithelial layer. Scale bar: 20 μm.

We detected the expression of LSS and its distribution in lens epithelial cells of the anterior pole and equator among WT, Lss^G589S/+^, and Lss^G589S/G589S^ mice at P0. In lens of WT mice, abundant LSS was mainly located in the lens epithelial layer adjacent to the EFI. In lens of Lss^G589S/+^ heterozygous mice, decreased LSS expression was observed in the lens epithelial layer, while its distribution was also mainly located in the lens epithelial layer adjacent to the EFI (Figure 3B, white arrows). In Lss^G589S/G589S^ homozygous mice, LSS was not located in the lens epithelial layer adjacent to the EFI and mainly diffused in the whole lens epithelial layer, as well as discontinuous EFI shown by F-actin staining (Figures 3A,B, red arrows). Thus, our finding showed that the distribution of lens LSS in Lss^G589S/G589S^ mice was distinctly different in WT and Lss^G589S/+^ heterozygous mice.

### Lens opacity in Lss^G589S/G589S^ mice was formed at the embryonic stage

**FIGURE 4:**
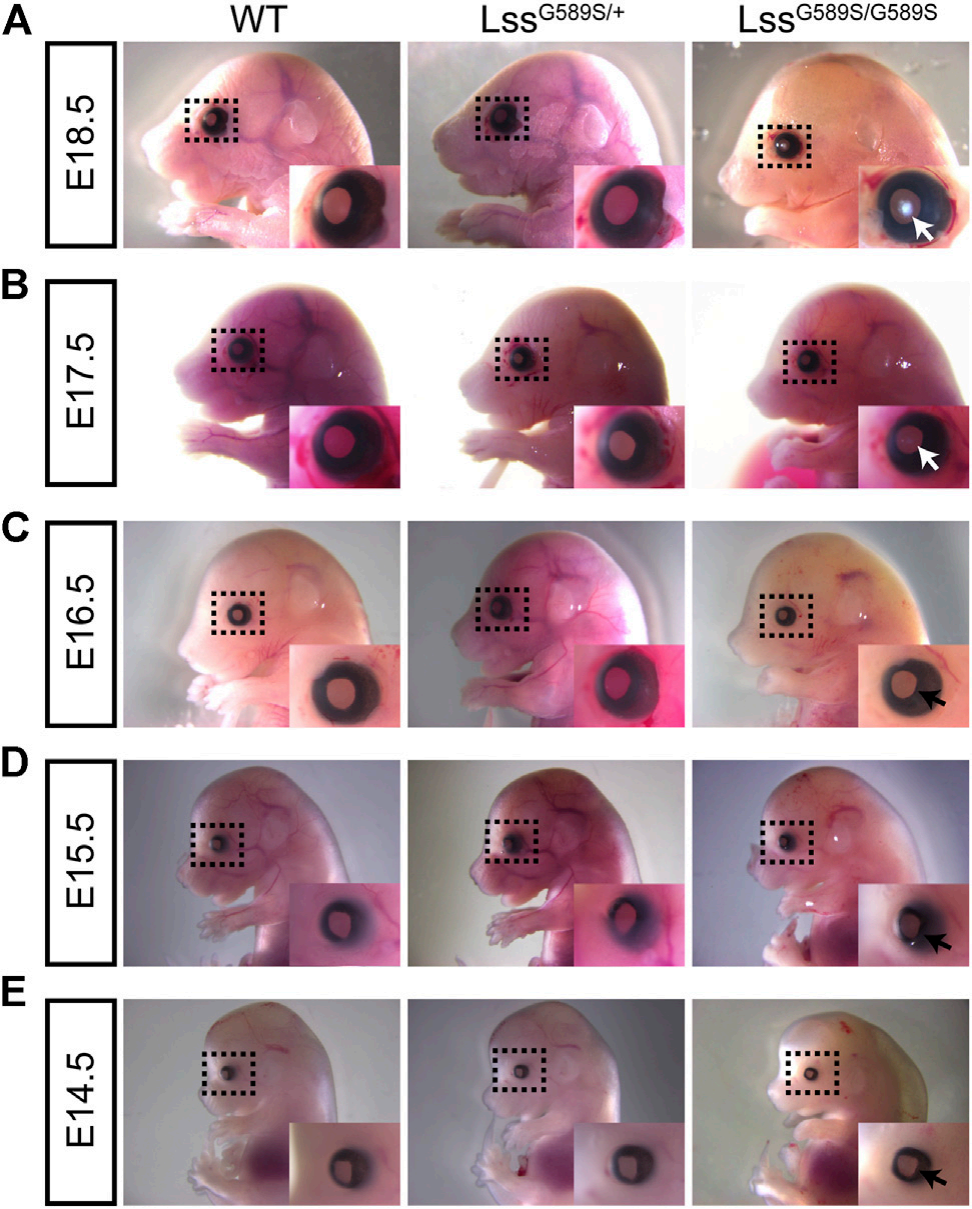
Lens opacity caused by LssG589S/G589S mutation was formed at the embryonic stage. Examination of lens transparency in WT, Lss^G589S/+^, and Lss^G589S/G589S^ mice at **(A)** E18.5, **(B)** E17.5, **(C)** E16.5, **(D)** E15.5, and **(E)** E14.5 by stereomicroscopy. White arrows indicate that mild cataract was formed at E17.5 and serious cataract was formed at E18.5 in the lens of Lss^G589S/G589S^ mice. Black arrows indicate that no visible cataract was observed at E14.5, E15.5, and E16.5 in the lens of Lss^G589S/G589S^ mice.

Our results above showed that the mouse model harboring a biallelic mutation of Lss G589S exhibited CC at P0; however, the time when the lens formed cataract is not clear. Thus, we investigated cataract formation time in Lss^G589S/G589S^ homozygous mouse lens at different embryonic stages (E14.5, E15.5, E16.5, E17.5, and E18.5). Ocular phenotypes of embryonic mice were observed by stereomicroscope. Our results demonstrated that mild cataract was formed at E17.5 (Figure 4B, E17.5, white arrow) and serious cataract was formed at E18.5 in the lens of Lss^G589S/G589S^ mice (Figure 4A, E18.5, white arrow). No visible cataract was observed at E14.5, E15.5, and E16.5 in the lens of Lss^G589S/G589S^ homozygous mice (Figures 4C–E, black arrow). Our findings indicated that visible opacity was formed in embryonic lens of Lss^G589S/G589S^ homozygous mice at E17.5.

### Abnormal morphology of fiber cells in embryonic lens of Lss^G589S/G589S^ mice

**FIGURE 5:**
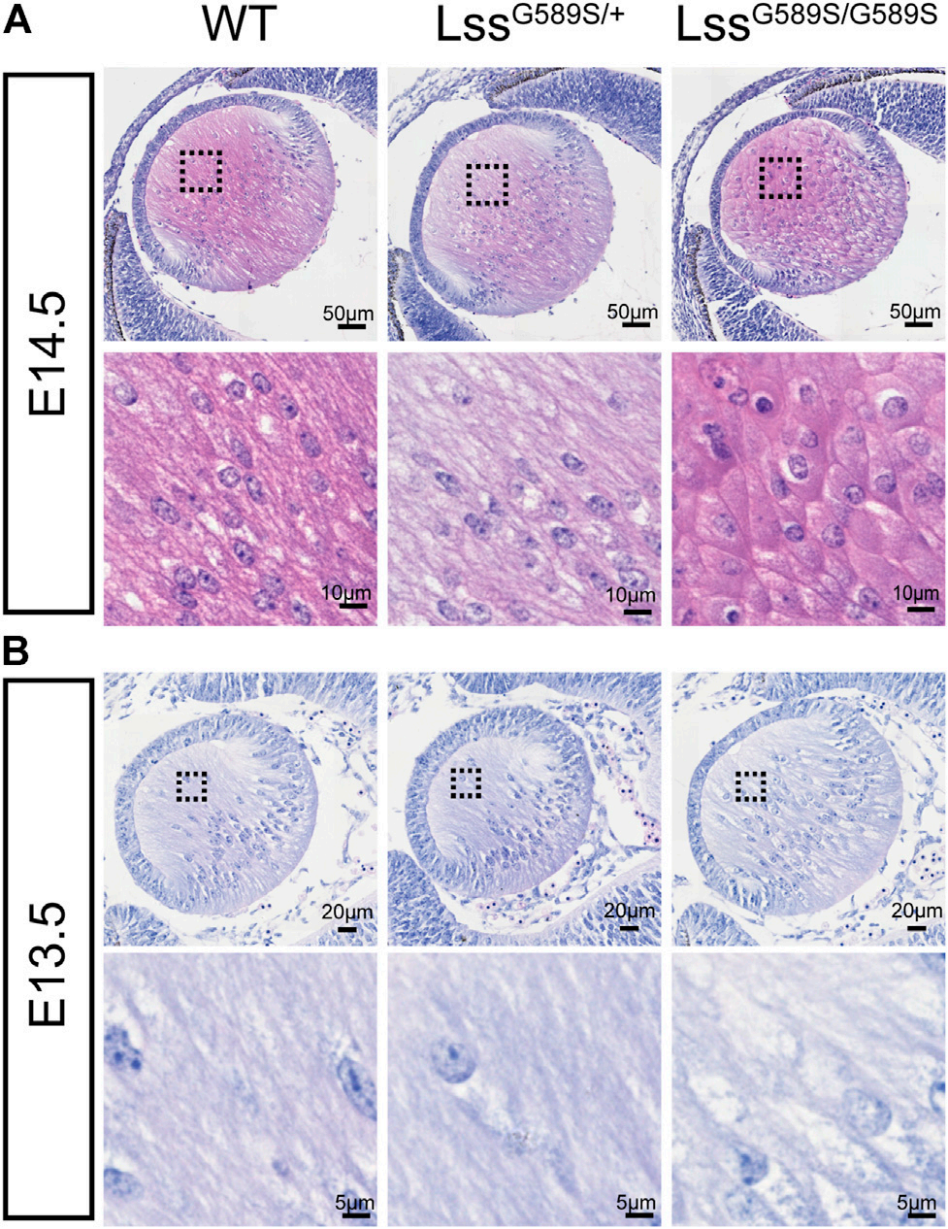
Morphology of fiber cells in embryonic lens of Lss^G589S/G589S^ mice. **(A)** Morphology of fiber cells stained with hematoxylin and eosin in embryonic lens of WT, Lss^G589S/+^, and Lss^G589S/G589S^ mice at E14.5. Scale bar: 50 μm. The enlargement is the magnification of the boxed inset shown in the upper panel. Scale bar: 10 μm. **(B)** Morphology of fiber cells in embryonic lens of WT, Lss^G589S/+^, and Lss^G589S/G589S^ mice at E13.5. Scale bar: 20 μm. The enlargement is the magnification of the boxed inset shown in the upper panel. Scale bar: 5 μm.

We then further investigated when structural defects were formed in embryonic lens of Lss^G589S/G589S^ mice. We found that refractive error could be observed in the lenses of Lss^G589S/G589S^ mice at E14.5, E15.5, and E16.5 after checking dissected eyeballs. Compared with fiber cells showing a long spindle shape in lens of WT and Lss^G589S/+^ heterozygous mice, fiber cells displayed an irregular rounded shape at the posterior pole in the lens of Lss^G589S/G589S^ homozygous mice at E14.5 (Figure 5A). At E13.5, a large part of fiber cells showed a normally elongated shape in the lens of Lss^G589S/G589S^ homozygous mice (Figure 5B). Our results showed that morphology of fiber cells was obviously abnormal in embryonic lens of Lss^G589S/G589S^ mice at E14.5.

### Altered expression and localization of Pax6 and Prox1 in lens fiber differentiation of Lss^G589S/G589S^ mice

**FIGURE 6:**
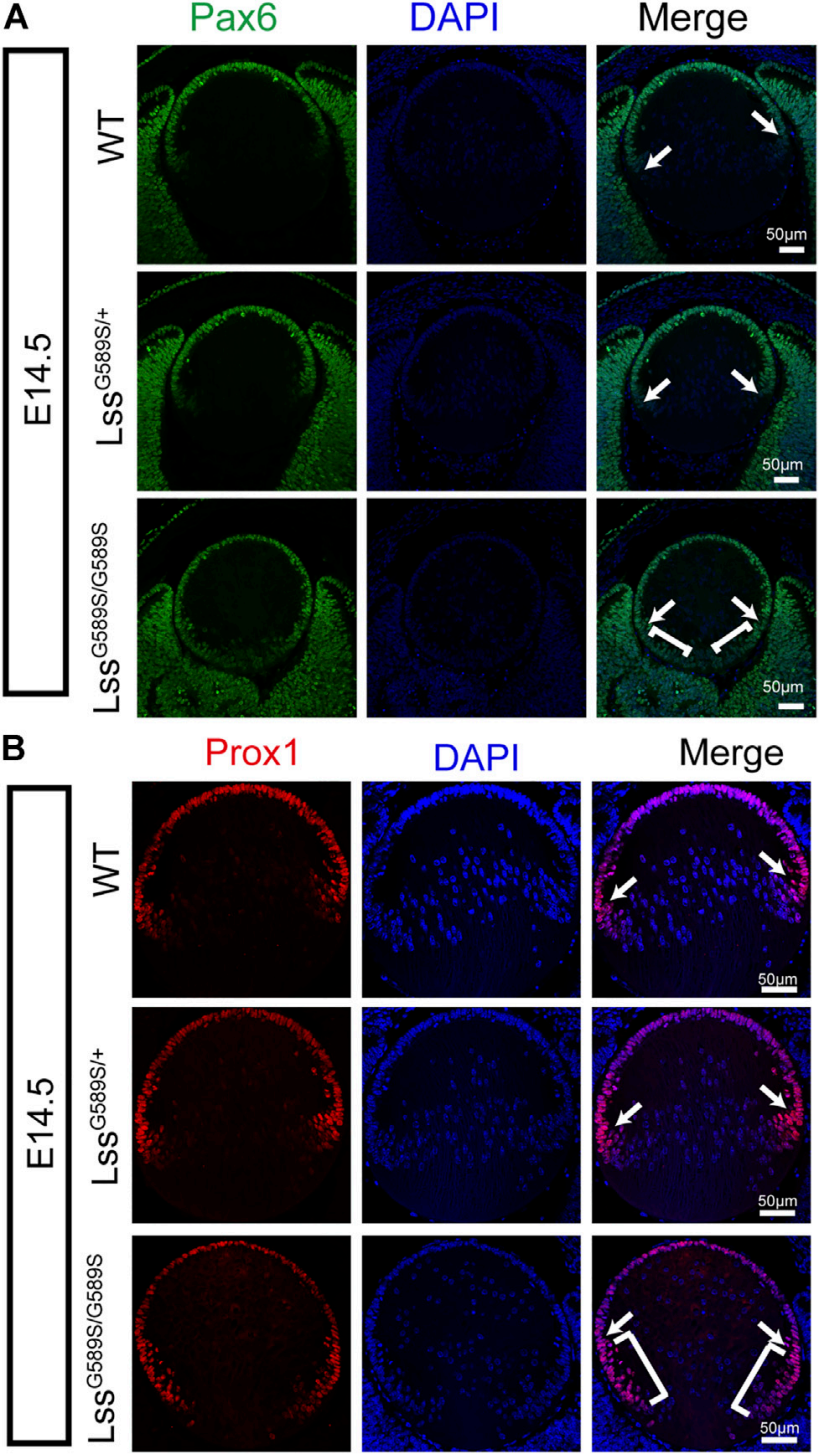
Altered expression and localization of Pax6 and Prox1 in lens epithelial cells of Lss^G589S/G589S^ mice at E14.5. **(A)** Expression and localization of Pax6 in lens epithelial cells of WT, Lss^G589S/+^, and Lss^G589S/G589S^ mice at E14.5. White arrows indicate that Pax6 was highly expressed in epithelial cells at the equator region of WT, Lss^G589S/+^, and Lss^G589S/G589S^ mice. White square brackets indicate that Pax6 was highly expressed at the transition zone and posterior terminals of lens in Lss^G589S/G589S^ mice. Scale bar: 50 μm. **(B)** Expression and localization of Prox1 in lens epithelial cells of WT, Lss^G589S/+^, and Lss^G589S/G589S^ mice at E14.5. White arrows indicate that Prox1 was highly expressed in epithelial cells at the equator region of WT, Lss^G589S/+^, and Lss^G589S/G589S^ mice. White square brackets indicate that Prox1 was highly expressed at the transition zone and posterior terminals of lens in Lss^G589S/G589S^ mice. Scale bar: 50 μm.

As abnormal lens development exhibited in Lss^G589S/G589S^ mouse embryos, we then investigated the expression of two transcription factors (Pax6 and Prox1) which are critical for normal differentiation of transparent lens in Lss^G589S/G589S^ embryos at E14.5. Pax6 plays a vital role in lens induction and fate determination, and Prox1 governs lens fiber cell differentiation and crystallin expression (Ashery-Padan et al., 2000; Cui et al., 2004; Audette et al., 2016; Collins et al., 2018). In our study, compared with WT and Lss^G589S/+^ heterozygous mice at E14.5, the expression and localization of Pax6 and Prox1 were significantly altered in the lens of Lss^G589S/G589S^ homozygous mice at E14.5 (Figures 6A,B). Most epithelial cells were still stained with Pax6 at the equator region (Figure 6A, white arrows) and posterior terminals of lens in Lss^G589S/G589S^ homozygous mice (Figure 6A, white square brackets). Also, Prox1 protein was highly expressed in epithelial cells not only at the equator region (Figure 6B, white arrow) but also at the transition zone and posterior terminals of lens in Lss^G589S/G589S^ homozygous mice (Figure 6B, white square brackets). These results revealed that an Lss G589S biallelic mutation led to significant change in the expression and localization of lens Pax6 and Prox1, which indicated that the process of lens fiber differentiation had been delayed in Lss^G589S/G589S^ homozygous mice at E14.5.

### Incomplete differentiation of lens fiber cells in Lss^G589S/G589S^ mice

**FIGURE 7:**
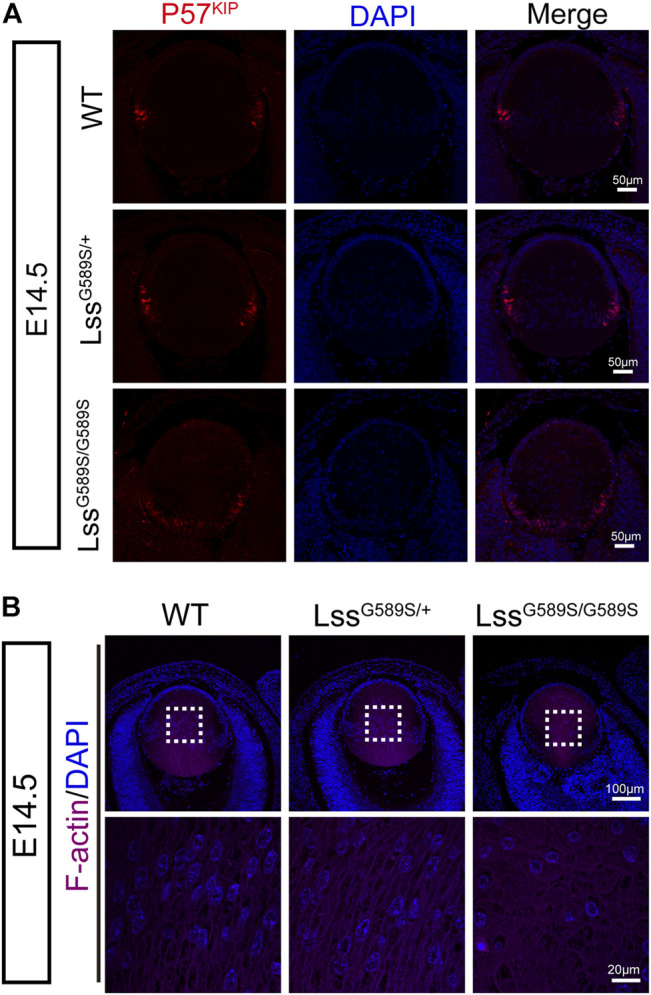
Incomplete differentiation of lens fiber cells in Lss^G589S/G589S^ mice. **(A)** The expression of p57^KIP2^ in WT, Lss^G589S/+^, and Lss^G589S/G589S^ mice lens at E14.5. Scale bar: 50 μm. **(B)** The distribution of lens fiber nuclei and alignment of lens fiber cells in WT, Lss^G589S/+^, and Lss^G589S/G589S^ mice at E14.5 using F-actin staining. Scale bar: 100 μm. The enlargement is the magnification of the boxed inset shown in the upper panel. Scale bar: 20 μm.

Cyclin-dependent kinase inhibitor p57^KIP2^, one of the downstream targets of Prox1, is required for the cells at lens equator withdrawal from the cell cycle and elongated to form fiber cells (Wigle et al., 1999; Shaham et al., 2009; Wiley et al., 2010). In Lss^G589S/G589S^ mice at E14.5, since Prox1 as a marker of fiber cell differentiation was strongly expressed in lens epithelial cells especially at the transition zone, we then assessed the expression and location of lens p57^KIP2^. In WT and Lss^G589S/+^ mice at E14.5, p57^KIP2^ was mainly located in lens epithelial cells at the lens equator (Figure 7A). In Lss^G589S/G589S^ mice at E14.5, more p57^KIP2^ was detected in lens epithelial cells at the region close to the posterior pole (Figure 7A). Moreover, the cells with nuclei are almost located at the regions from the lens equator to the anterior pole in WT and Lss^G589S/+^ mice at E14.5, while the cells with nuclei were seen almost throughout all the regions of lens in Lss^G589S/G589S^ mice at E14.5 (Figure 7B). In addition, F-actin staining revealed oval-shaped fiber cells with disordered arrangement located in the lens of Lss^G589S/G589S^ mice at E14.5, rather than elongated and well-organized fiber cells located in the lens of WT and Lss^G589S/+^ mice at E14.5 (Figure 7B). Thus, the Lss G589S homozygous mutation resulted in failure of epithelial cells at the lens posterior near the equator to exit the cell cycle and incomplete differentiation of lens fiber cells.

### Disrupted polarity of lens fiber cells in Lss^G589S/G589S^ mice

**FIGURE 8:**
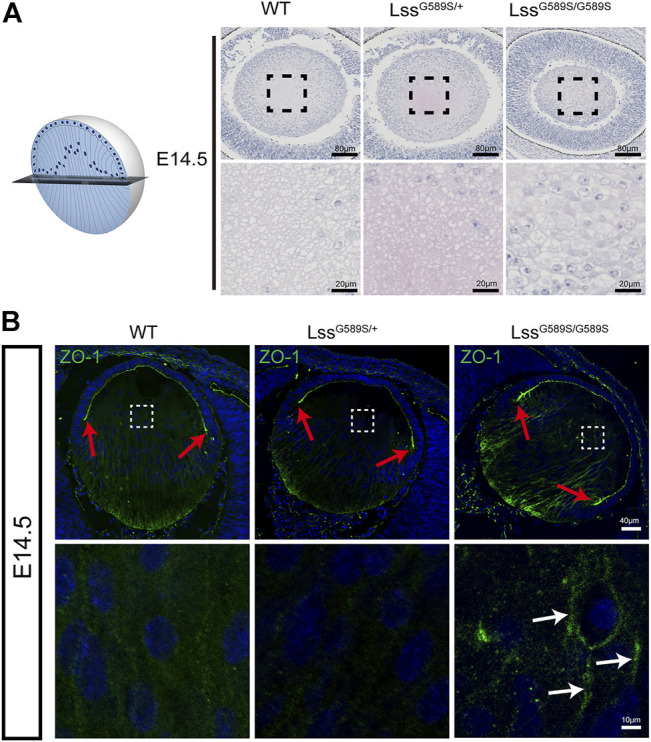
Disrupted polarity of lens fiber cells in Lss^G589S/G589S^ mice. **(A)** Alignment of lens fiber cells stained with hematoxylin and eosin and distribution of cell nuclei in sagittally sectioned lenses in the equator plane of WT, Lss^G589S/+^, and Lss^G589S/G589S^ mice at E14.5. Scale bar: 80 μm. The enlargement is the magnification of the boxed inset shown in the upper panel. Scale bar: 20 μm. **(B)** The expression of ZO-1 in lens of WT, Lss^G589S/+^, and Lss^G589S/G589S^ mice at E14.5. Scale bar: 40 μm. The enlargement is the magnification of the boxed inset shown in the upper panel. Red arrows indicate lens fulcrums. White arrow indicates that ZO-1 was strongly retained in the zones between lens fiber cells of Lss^G589S/G589S^ mice. Scale bar: 10 μm.

Our results above showed that F-actin was disorganized during fiber cell differentiation in Lss^G589S/G589S^ homozygous mice at E14.5. To further study the polarity of lens fibers at E14.5, lenses were sagittally sectioned in the equator plane (Figure 8A). At the equator plane, irregular size and swollen shape of fiber cells with plenty of nuclei were in the lens of Lss^G589S/G589S^ homozygous mice, while uniform size and round shape of fiber cells with a few nuclei were in the lens of WT and Lss^G589S/+^ heterozygous mice (Figure 8A). ZO-1, a tight-junction protein, has been reported to localize in the apical membrane of lens epithelial cells (indicated as the EFI) during the lens development stage (Nielsen et al., 2003; Wiley et al., 2010; Arora et al., 2012). In WT and heterozygous lenses at E14.5, ZO-1 was mainly expressed at the apical ends of the lens epithelial layer and prominently expressed at the lens fulcrum close to the equator (Figure 8B, red arrows). In Lss^G589S/G589S^ homozygous mice, ZO-1 was mainly expressed at the posterior fiber cell region and intensely expressed along the lens fulcrum (Figure 8B, red arrows); meanwhile, ZO-1 was strongly retained in the fiber zone between lens fiber cells (Figure 8B, white arrows). Taken together, our findings indicated that the lens fiber cells from homozygous mice lost their polarity in the apical–basal direction and failed to differentiate normally.

### Lss^G589S/G589S^ homozygous mutant resulted in a prominent disturbance of cholesterol biosynthesis pathways

**FIGURE 9:**
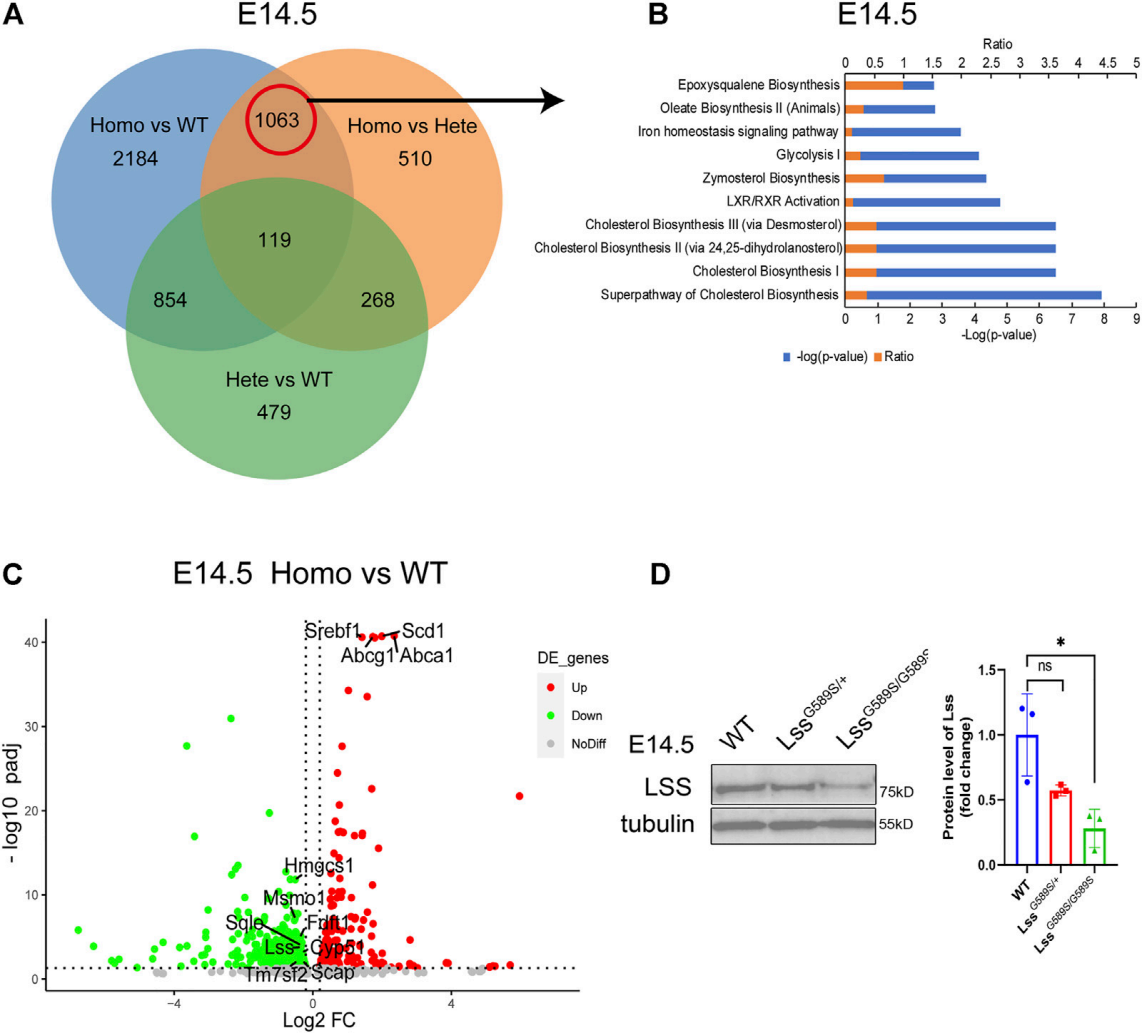
RNA-seq profiling in the lens of WT, Lss^G589S/+^, and Lss^G589S/G589S^ mice at E14.5. **(A)** Venn diagram analysis of differentially expressed genes (DEG) only overlapped in two groups (homozygous vs. WT, homozygous vs. heterozygous). **(B)** IPA pathway analysis of 1063 DEGs from the above results in **(A)**. Top 10 enriched canonical pathways were identified by applying the −log(*p*-value) > 2 threshold. Cholesterol biosynthesis pathways were significantly altered. **(C)** Volcano plot analysis showing downregulated and upregulated DEGs in the terms “cholesterol biosynthesis pathway” and “LXR/RXR pathway” (*p* adjust <0.05). **(D)** Western blot analysis of Lss from the lens of WT, Lss^G589S/+^, and Lss^G589S/G589S^ mice at E14.5. Anti-tubulin immunoblot was used as the reference. Statistics of Western blot **(right)**, *n* = 3 embryos per group. The data represent mean ± SEM. Significance was determined by two-tailed, unpaired Student’s t test. ns: no significance. *p < 0.05 versus WT group.

To explore potential mechanisms in lens development defects by the Lss G589S homozygous mutation, we performed transcriptomic profiling of lens collected from WT, Lss^G589S/+^, and Lss^G589S/G589S^ mice at E14.5. Due to normal lens development in wild-type and heterozygote mice, a Venn diagram was performed to analyze differentially expressed genes (DEGs) only overlapped in two groups (homozygous vs. WT, homozygous vs. heterozygous) by comparing DEGs in three groups (homozygous vs. WT, homozygous vs. heterozygous, and heterozygous vs. WT). It was found that 1063 DEGs were involved in the lens fiber differentiation process by comparing Lss^G589S/G589S^ homozygous mice with WT and Lss^G589S/+^ heterozygous mice (Figure 9A). The top 10 pathways were enriched by IPA (IPA) including cholesterol biosynthesis pathways and related metabolic pathways (Figure 9B). Several cholesterol biosynthesis genes such as Hmgcs1, methylsterol monooxygenase 1 (Msmo1), squalene epoxidase (Sqle), Lss, Fdft1, cytochrome P450 family 51 (Cyp51), Scap, and Tm7sf2 were significantly downregulated in the cholesterol biosynthesis pathway. Also, LXR/RXR pathway-related genes such as Abca1, Abcg1, Scd1, and Srebf1 were remarkably upregulated in lens from Lss^G589S/G589S^ mice (Figure 9C). Decreased expression of LSS in the lens of Lss^G589S/G589S^ mice at E14.5 was validated by Western blot analysis (Figure 9D). Herein, the downregulated cholesterol biosynthesis pathway caused by an Lss G589S homozygous mutation might partially account for lens development defects in cataractogenesis.

## Discussion

LSS is a cataract causal gene, and its mutations (G588S, W581R, I342S/W629C) result in CC (Mori et al., 2006; Zhao et al., 2015; Chen and Liu, 2017). Lanosterol synthase is the rate-limiting enzyme in the cholesterol biosynthesis pathway (Mori et al., 2006; Zhao et al., 2015) and crucial for maintenance of lens transparency *via* preventing abnormal protein aggregation (Zhao et al., 2015; Shen et al., 2018; Hua et al., 2019). Thus, it is important to investigate more functions of LSS. SCRs harboring hypomorphic Lss mutations and lens-specific Lss knockout mice were found to have cataracts at adult age (Mori et al., 2006; Wada et al., 2020); however, the roles of LSS during lens development remain largely unknown.

In our study, a mouse model harboring a Lss^G589S/G589S^ mutation (homologous to human LSS^G588S/G588S^ mutation) was generated to recapitulate CC. Similar to the phenotypes of human lens, there is no cataract found in the lens of WT and Lss^G589S/+^ heterozygous mice, while severe opacity was detected in the lens of Lss^G589S/G589S^ homozygous mice at P0, shown as disrupted EFI and disordered organization of lens fibers, and plenty of lens fiber nuclei were still retained in OFZ. Further studies on cataract formation at embryonic stages showed that visible cataracts formed in the embryonic stage at E17.5, and lens fibers failed to differentiate maturely at E14.5. Our study demonstrated that an Lss^G589S/G589S^ homozygous mutation resulted in disrupted lens structure and cataract at embryonic stages.

The main process of lens development includes induction, morphogenesis, differentiation, and growth (McAvoy et al., 1999; Collins et al., 2018). Many factors play an important role in lens epithelial proliferation and fiber differentiation (Cvekl and Zhang, 2017). Chromatin remodeling enzymes Brg1 and Snf2h regulate embryonic lens differentiation through the denucleation process (He et al., 2010; He et al., 2016). HSF4 regulated DLAD and promoted lens differentiation (Bu et al., 2002; Cui et al., 2013). CDK1 took part in nuclear removal during terminal lens fiber cell differentiation (Chaffee et al., 2014). Posttranslational modifications such as SUMOylation play fundamental roles in regulating lens differentiation (Nie et al., 2021). In our study, incomplete karyolysis (denucleation) was observed in the OFZ of lens in Lss^G589S/G589S^ homozygous mice at P0. Lss^G589S/G589S^ biallelic mutation might block lens primary fiber differentiation and subsequently lead to delayed differentiation of the secondary fiber. Thus, our results showed that LSS is required for lens fiber cell terminal differentiation and its denucleation.

Lens fiber cells maintain the same apical–basal polarity during the whole differentiation process of lens epithelial to fibers (Lo et al., 2000). The retained apical–basal polarity or apical cell junctions contribute to forming highly ordered and precisely aligned fiber cells, which is required for light transmission and lens transparency (Sugiyama et al., 2009). Lens-specific conditional knockout atypical protein kinase C (aPKC) was observed with disorganized fiber cell alignment (Sugiyama et al., 2009). Knockout of the neurofibromatosis type 2 (NF2) factor in lens caused lens cells to lose apical–basal polarity (Wiley et al., 2010). In our study, we found that Lss^G589S/G589S^ biallelic mutation caused a loss of apical–basal polarity in lens fiber cells and led to disturbed lens fiber differentiation at E14.5. Our results revealed that LSS is also required for the polarity of lens fiber during elongation.

LSS converts (S)-2,3-epoxysqualene to lanosterol in the cholesterol biosynthesis pathway, and lanosterol reverses protein aggregation in cataracts (Zhao et al., 2015; Wada et al., 2020). Lens-specific Lss knockout mice generated using Pax6-cre showed microphthalmia and small cloudy lenses (Wada et al., 2020). Also, the lens of tamoxifen-induced QKI knockout mouse formed cataract at P19 and developed a more severe cataract at P30 and exhibited a significantly downregulated cholesterol biosynthesis pathway including the Lss gene (Shin et al., 2021). Moreover, it is reported that lanosterol selectively stimulates HMGCR degradation and intermediates from the mevalonate pathway of cholesterol biosynthesis (Chen et al., 2019). In our study, Lss^G589S/G589S^ biallelic mutation led to a significantly decreased level of LSS protein and disturbed cholesterol synthesis pathways. Our RNA-seq profile of Lss^G589S/G589S^ lens showed that the expressions of Lss, Cyp51, Tm7sf2, Msmo1, Fdft1, and Sqle were significantly downregulated, which suggested that loss of function of LSS might disrupt the mevalonate pathway in regulating HMGCR degradation.

Lanosterol synthase is composed of two major domains at the N terminus (amino acid residues 84–325) and C terminus (amino acid residues 384–720) (Thoma et al., 2004; Romano et al., 2018). It is interesting that four LSS mutations causing CC are located toward the C terminus (Zhao et al., 2015; Chen and Liu, 2017), while five LSS mutation-causing autosomal-recessive hair loss disorders (hypotrichosis) are located toward the N terminus (Romano et al., 2018). Consequently, the mutations located at the C-terminus of LSS are more likely to cause cataract, whereas the mutations located at the N-terminus of LSS tends to cause hair loss. In addition, mutations of LSS can cause alopecia-mental retardation syndrome (APMR), which is a rare autosomal recessive neuro-dermal disorder (Muzammal et al., 2021). Thus, the studies on LSS mutations suggested that dysfunctions of LSS are far more complex and lead to autosomal-recessive diseases. It is also notable that two independent lines of Lss^G589S/G589S^ homozygous mice exhibited neonatal lethality at P0, which is distinctly different from humans as the patients are still alive at their teenage years. This means that Lss is required for mouse survival and the mice might be more sensitive to loss of function of Lss.

In summary, our study demonstrated that a mouse model harboring an Lss^G589S/G589S^ homozygous mutation can recapitulate human CC. Our findings confirmed loss of function of LSS disrupted differentiation and polarity of lens fibers in Lss^G589S/G589S^ mice. Thus, our study provides direct evidence that LSS plays an essential role in lens development, which will contribute to a better understanding of LSS functions in cataractogenesis and develop therapeutic approaches to cataracts.

## Data Availability

The datasets presented in this study can be found in online repositories. The names of the repository/repositories and accession number(s) can be found below: https://www.ncbi.nlm.nih.gov/geo/, GSE185143.
