# Supplementary material for: Defect of LSS Disrupts Lens Development in Cataractogenesis
Source: Front Cell Dev Biol. 2021 Dec 2;9:788422. doi: 10.3389/fcell.2021.788422 (PMC8675080; doi:10.3389/fcell.2021.788422)
Supplement: Supplementary file 1 [file DataSheet1.PDF]

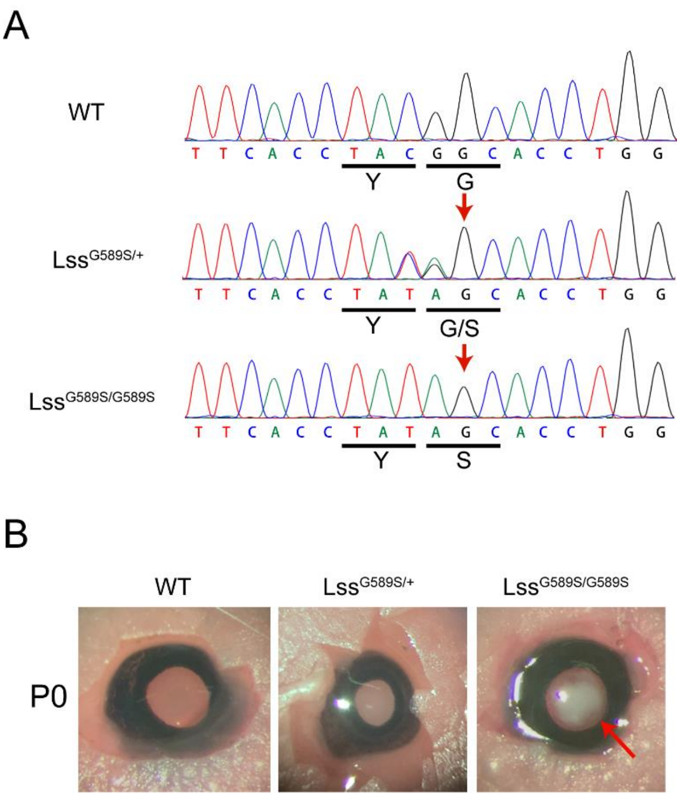

Supplemental Figure 1 Another line of Lss$^{G589S/G589S}$ showed congenital cataract.

A, Sanger sequencing was performed to validate the G589S missense mutation (GGC > AGC) in WT, Lss$^{G589S/+}$ and Lss$^{G589S/G589S}$ mice. Underlined sequences indicate the changed nucleic acids. Red arrows indicated G589S mutation site.

B, Mice lens were examined at P0. Red arrows indicated congenital cataract formed in lens of Lss$^{G589S/G589S}$ mice.

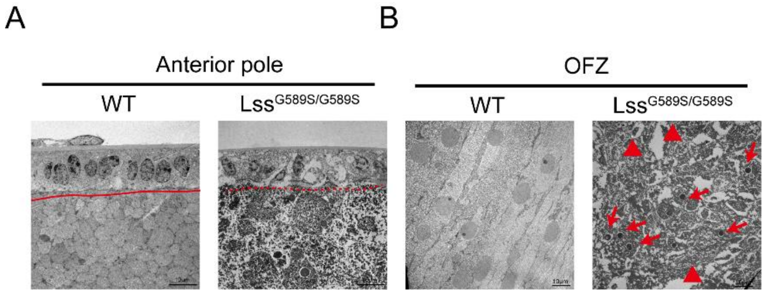

Supplemental Figure 2 Electron micrographs of lens morphology at anterior pole and OFZ regions in WT and Lss$^{G589S/G589S}$ mice.

A, At anterior pole region, representative images of lens epithelial-fiber interface (EFI) were shown. Red solid lines indicate lens EFI in WT mice. Red dashed line indicates lens EFI in Lss$^{G589S/G589S}$ mice. Scale bar: 10 μm.

B, At OFZ region, representative images of alignment and morphology of fiber cells were shown. Scale bar: 10 μm. Red triangle indicates high density deposits of fiber debris and red arrows indicate condensed nuclei in central OFZ of Lss$^{G589S/G589S}$ lens. Scale bar: 10 μm.
